# Dynamics of Forest Malaria Transmission in Balaghat District, Madhya Pradesh, India

**DOI:** 10.1371/journal.pone.0073730

**Published:** 2013-09-02

**Authors:** Neeru Singh, Sunil K. Chand, Praveen K. Bharti, Mrigendra P. Singh, Gyan Chand, Ashok K. Mishra, Man M. Shukla, Man M. Mahulia, Ravendra K. Sharma

**Affiliations:** 1 Regional Medical Research Centre for Tribals, RMRCT Campus, Nagpur Road, Post Garha, Jabalpur, Madhya Pradesh, India; 2 National Institute of Malaria Research Field Station, RMRCT Campus, Nagpur Road, Post Garha, Jabalpur, Madhya Pradesh, India; 3 Directorate of Health Services Madhya Pradesh, Satpuda Bhawan, Bhopal, Madhya Pradesh, India; National University of Singapore, Singapore

## Abstract

**Background:**

An epidemiological and entomological study was carried out in Balaghat district, Madhya Pradesh, India to understand the dynamics of forest malaria transmission in a difficult and hard to reach area where indoor residual spray and insecticide treated nets were used for vector control.

**Methods:**

This community based cross-sectional study was undertaken from January 2010 to December 2012 in Baihar and Birsa Community Health Centres of district Balaghat for screening malaria cases. Entomological surveillance included indoor resting collections, pyrethrum spray catches and light trap catches. Anophelines were assayed by ELISA for detection of Plasmodium circumsporozoite protein.

**Findings:**

*Plasmodium falciparum* infection accounted for >80% of all infections. *P. vivax* 16.5%, *P. malariae* 0.75% and remaining were mixed infections of *P. falciparum, P. vivax* and *P. malariae*. More than, 30% infections were found in infants under 6 months of age. Overall, an increasing trend in malaria positivity was observed from 2010 to 2012 (chi-square for trend  =  663.55; P<0.0001). Twenty five *Anopheles culicifacies* (sibling species C, D and E) were positive for circumsporozoite protein of *P. falciparum* (44%) and *P. vivax* (56%). Additionally, 2 *An. fluviatilis*, were found positive for *P. falciparum* and 1 for *P. vivax* (sibling species S and T). *An. fluviatilis* sibling species T was found as vector in forest villages for the first time in India.

**Conclusion:**

These results showed that the study villages are experiencing almost perennial malaria transmission inspite of indoor residual spray and insecticide treated nets. Therefore, there is a need for new indoor residual insecticides which has longer residual life or complete coverage of population with long lasting insecticide treated nets or both indoor residual spray and long lasting bed nets for effective vector control. There is a need to undertake a well designed case control study to evaluate the efficacy of these interventions.

## Introduction

Malaria is a global issue and India contributes substantially to global malaria incidence [1]. Tremendous progress has been made in the last decade in reducing malaria related morbidity and mortality in some countries partly because of increase in funding and partly because of new tools for malaria control such as long lasting insecticide treated nets (LLINs), rapid diagnostic tests (RDTs) and artemisinin based combination therapy (ACT) [2].

Factors influencing malaria in India are highly diverse and vary greatly from the epidemiological setting of any other country. The presence of various malarial parasites and vector species, climatic diversity favoring growth and proliferation of the parasite and vector as well as a highly susceptible human population have resulted in high malaria transmission in forested areas of central India (Madhya Pradesh). Indoor residual spraying (IRS) with synthetic pyrethroid and distribution of ACT were in use since 2008. Insecticide treated nets (ITNs) were introduced in this state since 2010. Despite these new tools, a very large number of malaria cases with high spleen rate were reported from district Balaghat, Madhya Pradesh [3], [4]. Understanding the multifaceted determinants of malaria transmission is important as many factors play a role in malaria transmission. A major challenge is the lack of knowledge about vectors, their seasonal abundance, infection rates and role in malaria transmission. Further, little information is available about the local patterns of malaria infection, the annual and seasonal changes in the prevalence with each *Plasmodium* species and the effect of antimalarial measures. Therefore, the objective of this study was to understand the dynamics of forest malaria transmission by conducting an epidemiological and entomological study in a difficult and hard to reach area where IRS, ITNs, RDTs and ACT were used for malaria control.

## Materials and Methods

### Study area and population

Balaghat district is a region of deep valleys, hills and hillocks with thick dense forest (longitude 80°15'E latitude 21°84'N, population 1756409). There are several perennial streams and their tributaries which provide numerous permanent breeding sites for mosquitoes. The villages are located near streams and most of the villages are unapproachable during rains (July - September).

**Figure 1:**
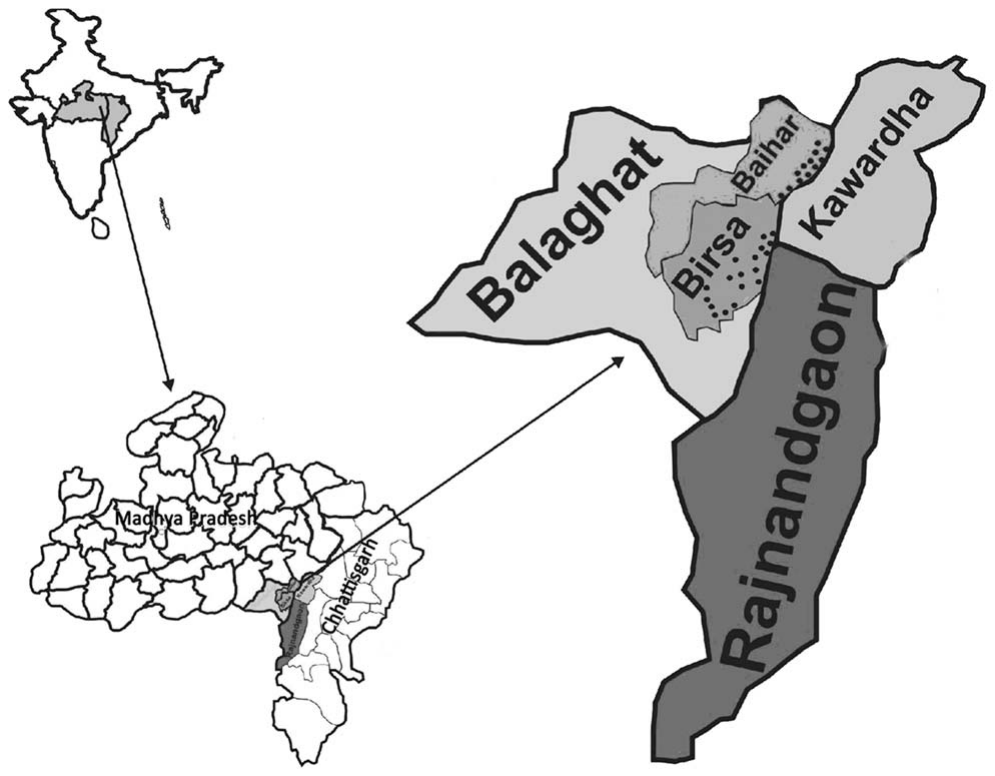
Map of India showing states of Madhya Pradesh and Chhattisgarh; District Balaghat and study villages in Baihar and Birsa community health centre. Also showing bordering districts Kawardha and Rajnandgaon of Chhattisgarh state.

This study was undertaken in 18 villages of Birsa (Population 11536) and 12 villages of Baihar (Population 6218) community health centres (CHCs) which are almost approachable throughout the year and on the border of Rajnandgaon and Kawardha districts of Chhattisgarh state (Figure 1). These villages are about 50–85 km from the state highway and are prone to frequent violence and disturbance [5]. The inhabitants of these villages are a particularly vulnerable tribal group called "Baigas" who generally have less access to education, skilled work and wealth creation opportunities. Overall literacy was 32% and among females literacy rate was only 20% [6]. The houses are made of woods and mud without ventilation and electricity. The local economy is mainly forest based, and agriculture is monsoon dependent. Most of the men and women work as labourers in forest nurseries, road construction and other casual jobs. These people spent most of their time outside the dwellings and sleep on the floor of the verandah (walled on 3 sides and open on one side) or outdoors during hot and humid months. Often domestic animals are sheltered in the house. The seasons are defined as spring (February - March), summer (April - June), monsoon (July - September), post monsoon (October - November) and winter (December - January). Vector control measures in this area included mainly two rounds of indoor residual spraying (IRS) of dwelling with a synthetic pyrethroid (alphacypermethrin). In July – August 2010 insecticide treated bed nets (ITNs) were also given for the first time in these villages (2 per family). The study area is under Enhanced Malaria Control Project with World Bank assistance.

Before undertaking this investigation, data from previous years of the district were obtained from District Malaria Officer (DMO), Balaghat along with details of insecticide used for spray, population covered, types of antimalarial used and their numbers and RDTs used etc (Table 1, 2). The sample size was calculated on the basis of surveillance data available from Birsa and Baihar CHCs as per records of DMO, Balaghat. The average SPR for last three years (2007-09) for Birsa and Baihar CHCs was 1.61%. Thus assuming average SPR as 1.61% for study area and a relative precision of 30% (i.e. estimated SPR will vary between 1.27–2.09%), and level of confidence as 95%, the minimum required sample size was 2609 for blood smear collection.

**Table 1:** Indoor residual insecticide spray (IRS) of CHC Birsa and Baihar, District Balaghat.*

Year	No of villages	Population	(%) Coverage		Insecticide consumption (kg)		
			House	Room	DDT (50%)	Alpha cypermethrin (5%)	Lambda cyhalothrin (10%)
2007	108	80556	95.4	89.1	5583	1530	-
2008	103	96004	95.2	87.2	-	3340	-
2009	50	39272	97.2	90.5	-	1406	-
2010	182	177439	96.8	89.2	-	-	3300
2011	64	46547	92.0	81.0	-	1800	70
2012	224	197399	95.5	85.2	-	6970	-

* Source: District Malaria Officer, Balaghat.

**Table 2:** Drug/diagnostic consumption of District Balaghat.*

Year	Cloroquine (CQ)	Suphadoxine pyrimethamine (SP)	Primaquine		Rapid diagnostic kits (Pf)	Artesunate + Suphadoxine pyrimethamine (ACT)	Blister combipack (Chloroquine + Primaquine)
			7.5 mg	2.5 mg			
2007	862973	700	254982	74715	61160	0	169696
2008	1087367	3600	288364	180418	38303	13350	127386
2009	1187172	10725	380054	311874	23757	9150	59505
2010	1390842	24550	312050	269827	28275	100000	6110
2011	1361221	4225	127098	30393	18650	11596	0
2012	1180493	0	14711	0	142771	3538	0

* Source: District Malaria Officer, Balaghat.

### Epidemiological surveillance

Monthly cross sectional fever surveys were carried out door-to-door in study villages from January 2010 to December 2012. Finger-pricked blood smears were collected from all fever cases and cases with 14 days history of fever as per the guidelines of National Vector Borne Disease Control Programme (NVBDCP). The blood smears thus collected were stained with the JSB stain [7]. The microscopist examined 100 fields in thick smears under 1000x magnification before declaring it negative. For quality control 100% of positive smears and 10% of negative smears were re-examined by second expert who was unaware of previous results.

Spleen examination was done in children between 2–9 years with or without fever by Hackett’s method [8]. All parasite positive *P. falciparum* cases were given ACT [Artesunate Combination Therapy (Artesunate + Sulfadoxine Pyrimethamine)] and Primaquine (PQ) and all *P. vivax* cases were given Chloroquine (CQ) and PQ as per NVBDCP guidelines [9]. Infants and pregnant women were not given PQ.

### Therapeutic efficacy studies

Overall 105 patients (aged 1–59 years) were enrolled with ACT orally over a three-day period. Clinical observations were recorded daily for the first eight days (0–7 days) and during follow- up following standard protocol [10]. Blood smears were prepared from each patient on days 0, 1, 2, 3, 7, 14, 21 and 28 days and examined by light microscopy to monitor the parasitological response. Finger prick blood samples were also collected on filter paper for molecular study. The therapeutic efficacy outcome was determined for 77 patients and 28 patients did not complete the study.

### Entomological surveillance

Indoor resting Anopheles mosquitoes per man hour (MHD) were sampled once in a month in the early morning (0600 hrs) for 15 minutes each by a team of two insect collectors with flashlights and mouth aspirators in four villages (two from each CHC) as per standard techniques [11]. Pyrethrum spray catches (PSC) were made once in a month from human dwellings (HD) randomly selected other than those selected for indoor resting collection from two villages (one village from each CHC) between 0600 hour to 0930 hour. Light trap catches (LT) indoor-outdoor were also made once in a month in two villages (one from each CHC) other than those selected for MHD and PSC as described earlier [12]. Human bait catches were not performed owing to ethical reasons.

Insecticide susceptibility tests were carried out on field collected *An. culicifacies* using DDT (4%), malathion (5%) and deltamethrine (0.05%) test papers following standard techniques [13]. The efficacy of alphacypermethrin was determined by cone bioassay method. Anophelines were assayed by ELISA for detection of Plasmodium circumsporozoite protein (CSP). The ELISA protocol described by Wirtz *et al*. (1987) [14] was followed to detect CSP of *P. falciparum* and *P. vivax*. PCR and D3 domain of 28S rDNA gene sequencing was used to identify all specimens of the *An. culicifacies* and *An. fluviatilis* species complex as described earlier [15].

### Data analysis

The data were double key-entered in MS Access 2003 (Microsoft Corporation, Redmond, Washington, USA) based data entry screen. Age was recoded in categorical variable (up to 1, >1–4, >4–8, >8–14 and above 14 years of age) before analysis. 2×2 contingency table analyses were used to describe the differences in case prevalence by age groups. Data analysis was done using SPSS 17 for Windows (SPSS Inc., Chicago, Illinois).

The slide positivity rate (SPR) is the proportion of examined thick films found parasite positive and the related slide falciparum rate (SFR) and slide vivax rate (SVR) were calculated. Gametocyte carriers were recorded only for *P. falciparum*.

### Ethics

Informed written consent was obtained from each adult and from guardian of the children enrolled as per ethical guidelines of Indian Council of Medical Research, New Delhi, India. Purpose, potential risk and benefits of the study has been communicated in local language (‘Hindi’) and consent was documented, even for those who were unable to read and write by taking thumb impression in the presence of a witness specifically from the same community. Generally school teachers, forest guards and other influential members of their community are very helpful in explaining benefit of the study and obtaining consent. The study was approved by the institutional review board of Regional Medical Research Centre for Tribals, Jabalpur, India (IRB00006471).

## Results

### Epidemiological studies

**Figure 2:**
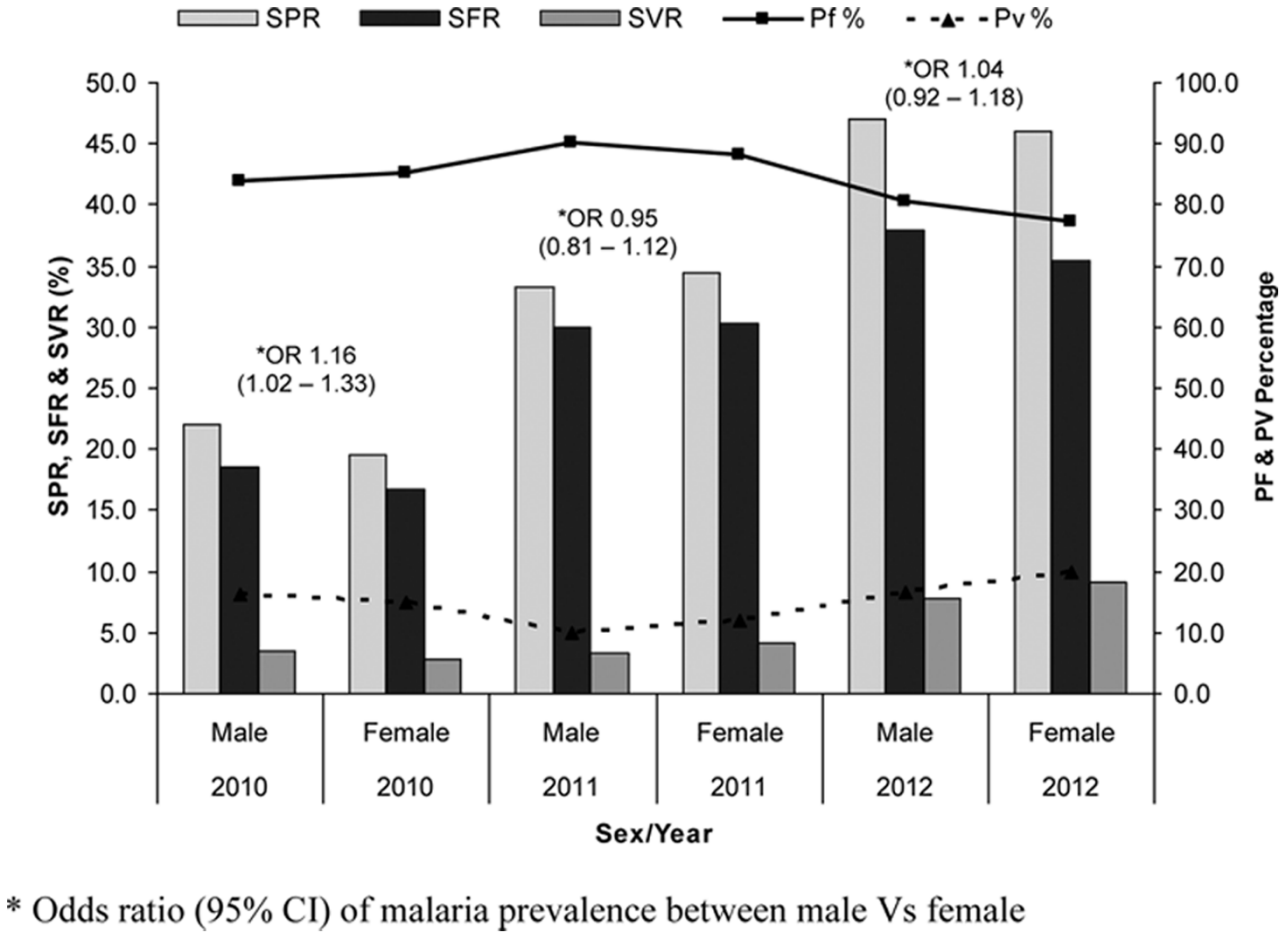
Sex wise malaria prevalence in study villages of Balaghat district (2010 – 2012).

Of the total 5683 males and 5939 females screened for malaria, 1881 males (33%) and 1868 females (31.5%) were positive for malaria (OR 1.08, 95% CI 1.0–1.16). Figure 2 showed sex wise malaria positivity rates from 2010 to 2012. Age-specific and species specific data on malaria are shown in Table 3. The malaria positivity rates varied over the years and *P. malariae* was found only in 2012.

**Table 3:** Year/age group wise malaria prevalence in rapid fever survey in CHC Birsa and Baihar, District Balaghat (2010 – 2012).

Year	Age group (years)	+ve/BSE	Pf	Pv	Pm	Mixed (Pf+Pv)	Mixed (Pm+Pf/Pv)	SPR	SFR	SVR	Pf g rate	OR (malaria)	95% CI
2010	≤1	49/189	30	19	0	0	0	25.9	15.9	10.1	26.7	2.9	2.0–4.1***
	>1–4	217/708	167	49	0	1	0	30.6	23.7	6.9	15.5	3.6	2.9–4.5***
	>4–8	296/1015	248	48	0	0	0	29.2	24.4	4.7	8.1	3.4	2.8–4.1***
	>8–14	267/1135	240	27	0	0	0	23.5	21.1	2.4	5.8	2.5	2.1–3.1***
	>14	219/2011	200	19	0	0	0	10.9	9.9	0.9	10.0	1.0	Reference
	**Total**	**1048/5058**	**885**	**162**	**0**	**1**	**0**	**20.7**	**17.5**	**3.2**	**9.9**		
2011	≤1	59/222	43	16	0	0	0	26.6	19.4	7.2	9.3	1.2	0.9–1.7***
	>1–4	160/331	121	35	0	4	0	48.3	37.8	10.6	16.8	3.1	2.4–4.0***
	>4–8	259/519	236	21	0	2	0	49.9	45.9	4.0	14.3	3.3	2.6–4.1***
	>8–14	221/672	202	19	0	0	0	32.9	30.1	2.8	7.4	1.6	1.3–2.0***
	>14	242/1037	231	11	0	0	0	23.3	22.3	1.1	6.1	1.0	Reference
	**Total**	**941/2781**	**833**	**102**	**0**	**6**	**0**	**33.8**	**30.2**	**3.7**	**10.5**		
2012	≤1	70/142	41	23	0	5	1	49.3	32.4	16.2	23.9	2.7	1.9–3.9***
	>1–4	291/451	185	70	4	24	8	64.5	46.3	15.5	28.2	5.1	3.9–6.6***
	>4–8	501/861	362	91	16	25	7	58.2	44.9	10.6	25.6	3.9	3.1–4.8***
	>8–14	680/1504	543	111	7	11	8	45.2	36.8	7.4	14.8	2.3	1.9–2.8***
	>14	218/825	190	25	2	1	0	26.4	23.2	3.0	10.5	1.0	Reference
	**Total**	**1760/3783**	**1321**	**320**	**29**	**66**	**24**	**46.5**	**36.7**	**8.5**	**19.5**		

*** P<0.001; +ve: Malaria parasitaemic cases; BSE: Blood slide examined; Pf: *Plasmodium falciparum*; Pv: *Plasmodium vivax*; Pm: *Plasmodium malariae*; SPR: Slide positivity rate; SFR: Slide falciparum rate; SVR: Slide vivax rate; Pfg rate: *Plasmodium falciparum* gametocytes rate; OR (malaria): Odds ratio for malaria positive; 95% CI: 95% confidence interval.

The SPR was lowest in adults (aged >14 yrs) when compared with other age groups (OR 3.1; 95% CI 2.8–3.4) (P< 0.0001). The SVR was highest in infants < = 1 year) (OR 8.1; 95% CI 5.5–12.0) when compared with adults (P<0.001) (Table 3). Further analysis revealed that out of 178 infants positive for malaria, 58 were less than 6 months (33%) of which 40 were positive for *P. falciparum* and 18 for *P. vivax*. However, SFR was highest in relatively older children > 4–8 years (OR 3.0; 95% CI 2.7–3.4) compared with adults (P<0.001). *P. falciparum* proportion was higher in all age groups as compared to *P. vivax* (P<0.001). The mean gametocyte rate was 14.4% and monitoring of gametocyte prevalence revealed that young children less than 5 years have significantly more gametocytes as compared to relatively older children (>5 years and above) (OR 1.8; 95% CI 1.4–2.3).

Further analysis of the distribution of malaria cases by season revealed that SFR was highest in post monsoon season 40.3% (OR, 2.9; 95% CI 2.6–3.3) and lowest in summer 18.7% (Table 4). The gametocyte rate was highest in spring (OR 3.9; 95% CI 2.6–5.8) and lowest in monsoon while SVR was highest in summer (OR 1.4; 95% CI 1.1–1.8) and lowest in post monsoon. Examination of blood smears in children with enlarged spleen revealed that highest falciparum prevalence was observed in post monsoon months as compared to spring (OR 4.1; 95% CI 3.0–5.5) while *P. vivax* prevalence was highest in summer (OR 3.2; 95% CI 1.9–5.2). Spleen rate was 48.5% in 2010 which increased to 58.3% in 2012 (OR 1.5; 95% CI 1.3–1.7). The average spleen rate in children was 43.8% (2269/5182) and the average enlarged spleen was 1.99 (95% CI 1.94–2.04). Year wise analysis revealed (Table 3) that SPR increased from 20.7% in 2010 to 33.8% in 2011 (i.e. 13.1 point increase) and further 46.6% in 2012 (an increase of 12.8 points between 2011-12). Similarly SFR increased from 17.5 in 2010 to 30.2 (12.7 point increase) and further 36.7 in 2012 (an increase of 6.5 points between 2011-12). However, the proportion of *P. falciparum* declined by 5.7% in 2012 when compared with 2010.

**Table 4:** Year/season wise malaria prevalence in CHC Birsa and Baihar, District Balaghat (2010 – 2012).

Year	Seasons	+ve/BSE	Pf	Pv	Pm	Mixed (Pf+Pv)	Mixed (Pm+Pf/Pv)	SPR	SFR	SVR	Pf g rate	OR (malaria)	95% CI
2010	Spring (Feb-Mar)	102/1051	86	16	0	0	0	9.7	8.2	1.5	14.0	1	Reference
	Summer (Apr-Jun)	242/1359	177	65	0	0	0	17.8	13.0	4.8	11.3	2.0	1.6–2.6^***^
	Monsoon (Jul-Sep)	210/705	190	20	0	0	0	29.8	27.0	2.8	7.4	3.9	3.0–5.2^***^
	Post monsoon (Oct-Nov)	178/832	155	23	0	0	0	21.4	18.6	2.8	8.4	2.5	1.9–3.3^***^
	Winter (Dec-Jan)	316/1111	277	38	0	1	0	28.4	25.0	3.4	10.4	3.7	2.9–4.7^***^
	**Total**	**1048/5058**	**885**	**162**	**0**	**1**	**0**	**20.7**	**17.5**	**3.2**	**9.9**		
2011	Spring (Feb-Mar)	78/501	63	15	0	0	0	15.6	12.6	3.0	6.3	1	Reference
	Summer (Apr-Jun)	182/811	156	26	0	0	0	22.4	19.2	3.2	1.9	1.6	1.2–2.1^**^
	Monsoon (Jul-Sep)	56/251	48	7	0	1	0	22.3	19.5	2.8	2.0	1.6	1.1–2.3*
	Post monsoon (Oct-Nov)	473/698	432	38	0	3	0	67.8	62.3	5.4	9.2	11.4	8.1–16.0^***^
	Winter (Dec-Jan)	152/520	134	16	0	2	0	29.2	26.2	3.1	29.4	2.2	1.6–3.1^***^
	**Total**	**941/2781**	**833**	**102**	**0**	**6**	**0**	**33.8**	**30.2**	**3.7**	**10.5**		
2012	Spring (Feb-Mar)	521/993	325	103	23	46	24	52.5	37.4	10.4	29.1	1	Reference
	Summer (Apr-Jun)	279/663	188	76	5	10	0	42.1	29.9	11.5	15.7	0.7	0.5–0.8^***^
	Monsoon (Jul-Sep)	289/752	226	63	0	0	0	38.4	30.1	8.4	8.8	0.6	0.5–0.7^***^
	Post monsoon (Oct-Nov)	386/799	345	38	0	3	0	48.3	43.6	4.8	18.7	0.8	0.7–1.0*
	Winter (Dec-Jan)	285/576	237	40	1	7	0	49.5	42.4	6.9	19.3	0.9	0.7–1.1^**^
	**Total**	**1760/3783**	**1321**	**320**	**29**	**66**	**24**	**46.5**	**36.7**	**8.5**	**19.5**		

* P<0.05; ^**^P<0.01; ^***^P<0.001; +ve: Malaria parasitaemic cases; BSE: Blood slide examined; Pf: *Plasmodium falciparum*; Pv: *Plasmodium vivax*; Pm: *Plasmodium malariae*; SPR: Slide positivity rate; SFR: Slide falciparum rate; SVR: Slide vivax rate; Pfg rate: *Plasmodium falciparum* gametocytes rate; OR (malaria): Odds ratio for malaria positive; 95% CI: 95% confidence interval.

Over all therapeutic efficacy study showed 100% adequate clinical and parasitological response and follow up of patients up to 28 days did not show clinical symptoms and malaria parasites.

### Entomological studies

**Figure 3:**
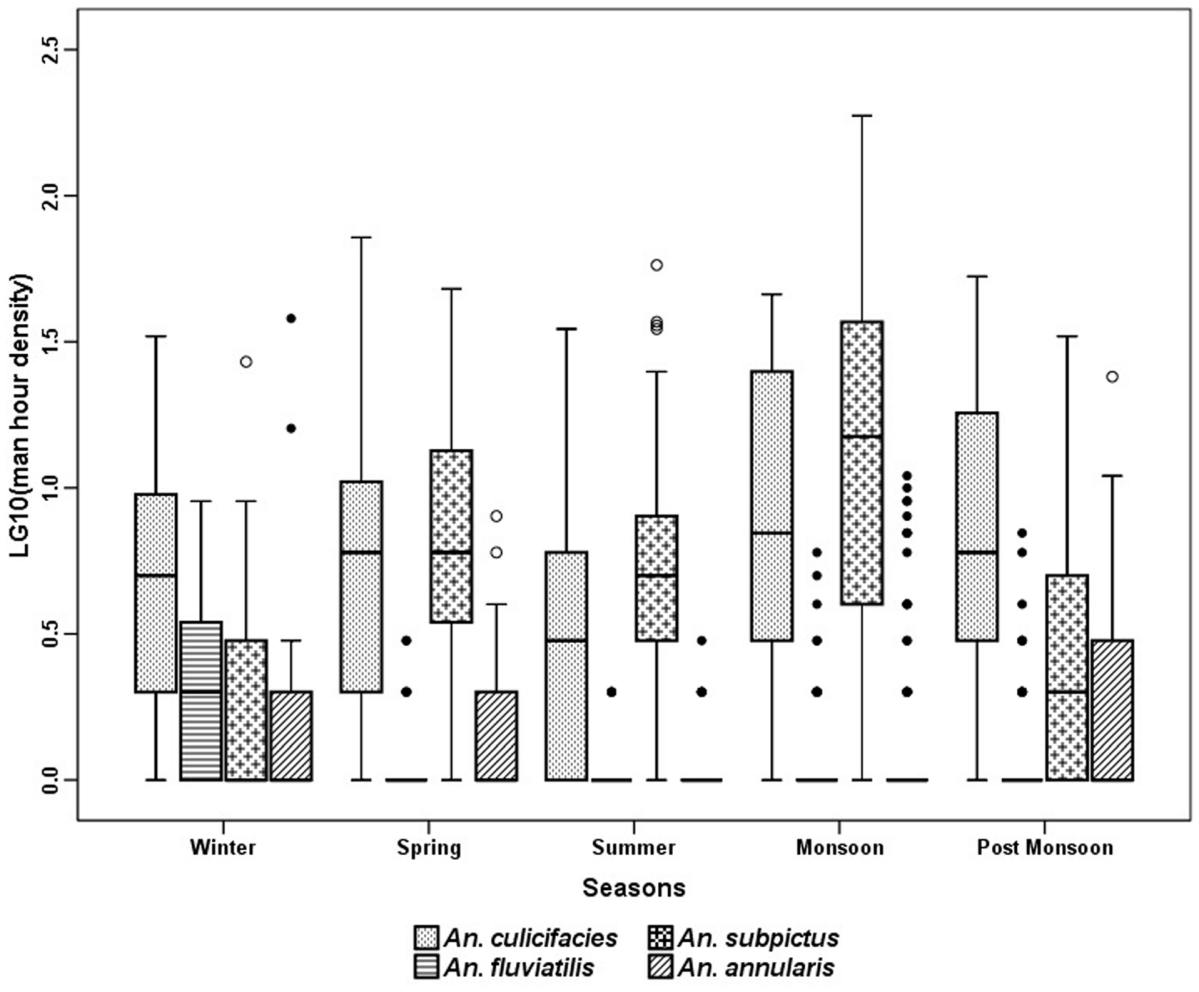
Season wise average per man hour density (indoor resting collection) in villages of Balaghat (2010-2012).

**Figure 4:**
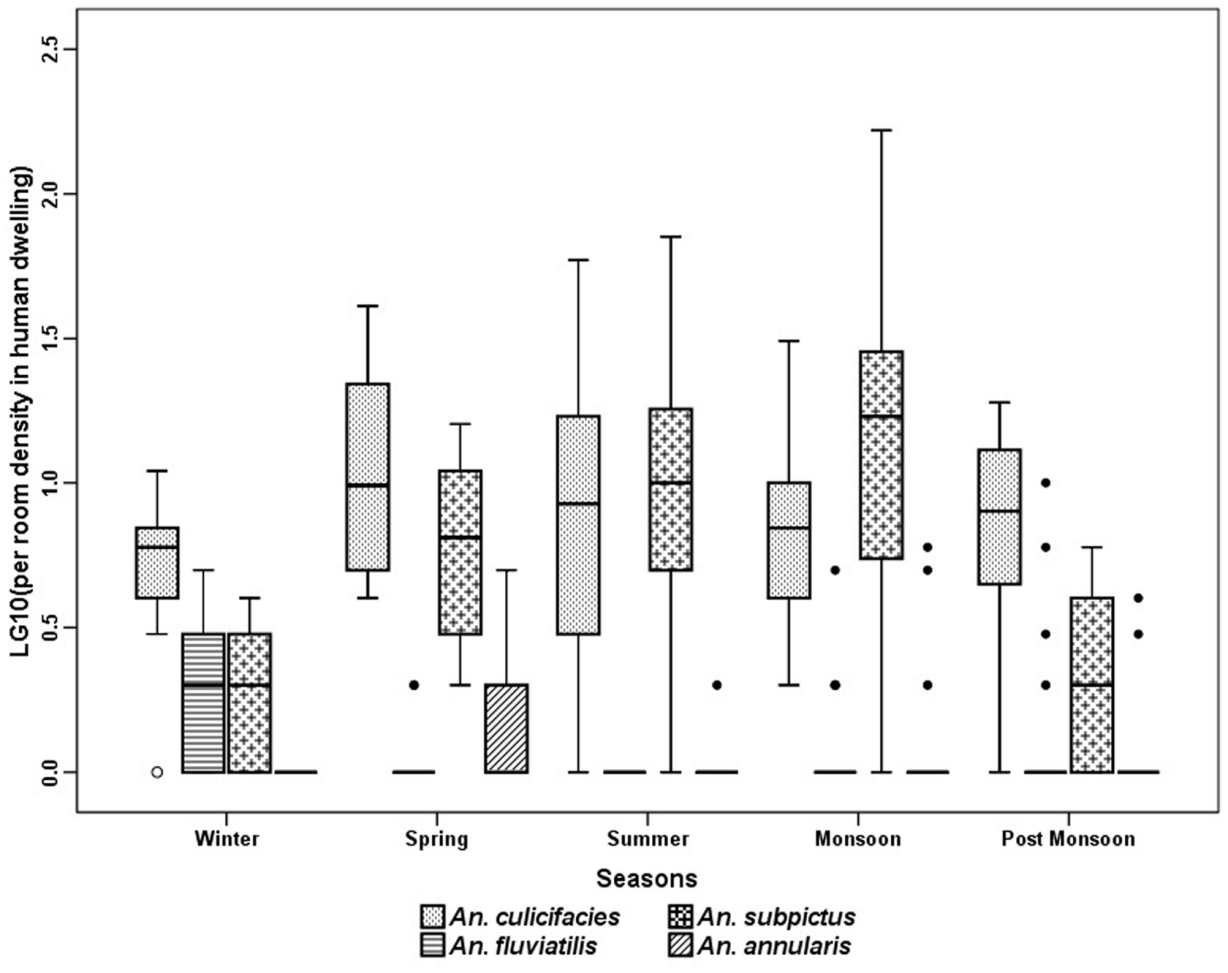
Season wise pyrethrum spray catches in human dwelling in Balaghat (2010-2012).

**Figure 5:**
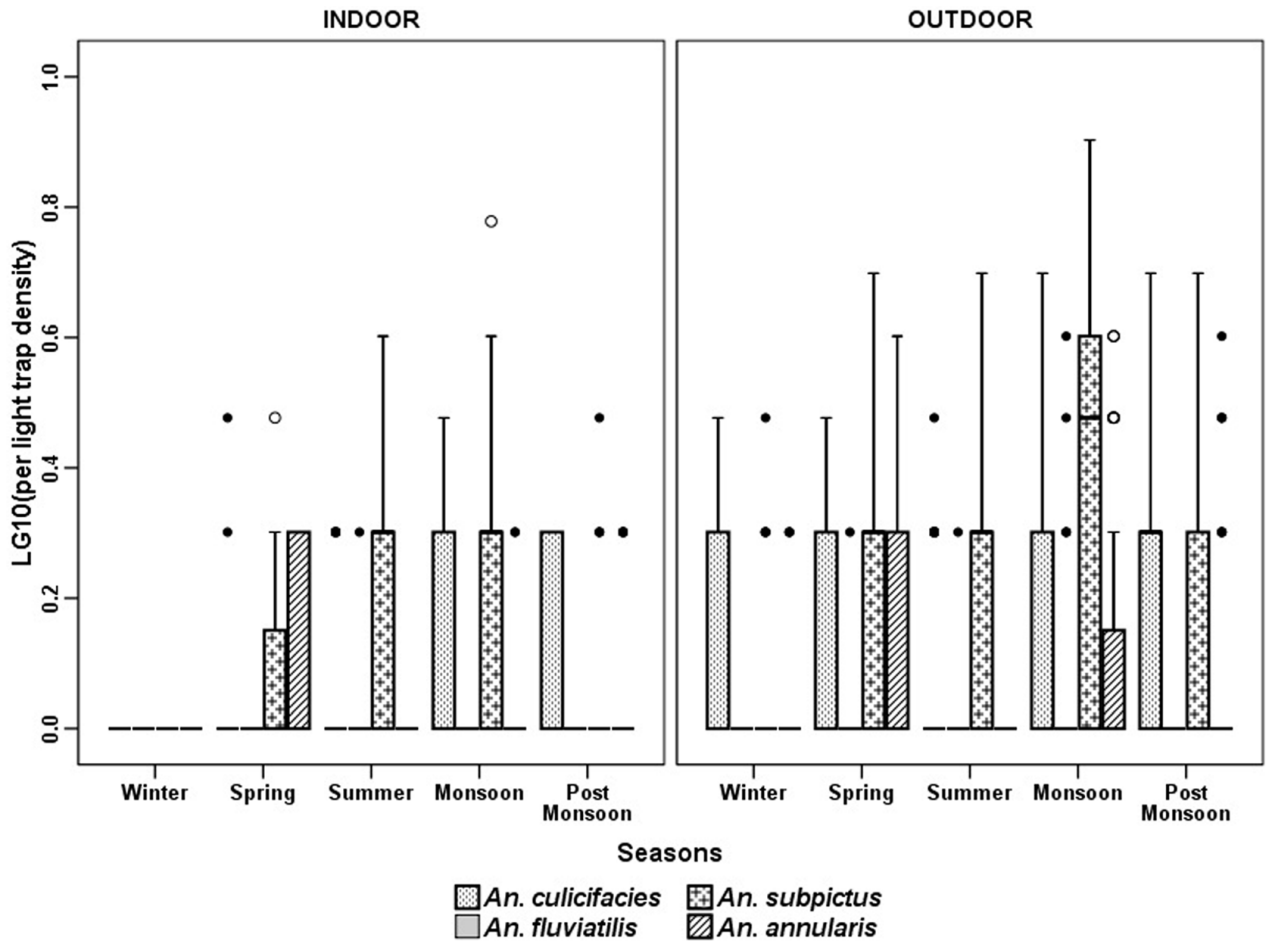
Season wise light trap catches (indoor and outdoor trap collection) in villages of Balaghat (2010-2012).

The anopheline fauna of the villages consisted of 9 species, of which *An. culicifacies* Giles, *An. subpictus* Grassi, *An. fluviatilis* James, *An. annularsis* Van Der Wulp were the most abundant species in indoor resting collection (MHD) (Figure 3). *An. vagus* Donitz, *An. pallidus Theobald*, *An. barbirostris* Van Der Wulp. *An. nigerrimus* Giles and *An. jeyporiensis* Froud were collected in very small numbers. The number of anopheline mosquitoes collected throughout the year did not show a consistent pattern. The 301 man hour of efforts revealed that *An. subpictus* was the most predominant species (53.5%) with a peak in monsoon months. The relative abundance of *An. culicifacies* was high throughout the year (39%) with a peak in post monsoon season (October - November). *An. fluviatilis* was collected mainly in winter season (December - January) and was almost absent during the dry hot months of April to June. The trend of anopheline prevalence was more or less similar in MHD (Figure 3) and PSC (Figure 4) except in case of *An. annularsis* which was found relatively more in MHD collections in post monsoon and winter seasons compared to respective PSC catches in these seasons. Three more species were found in LT in very small numbers. These are *An. splendidus* Koidzumi, *An. theobaldi* Giles and *An. tessellates* Theobald. Comparison of season wise catches of light trap revealed that significantly more *An. culicifacies* were found in post monsoon and winter seasons, *An. fluviatilis* in monsoon season, *An. subpictus* in monsoon, post monsoon and winter and *An. annularsis* were found during monsoon in outdoor catches as compared to indoor (Figure 5).

**Figure 6:**
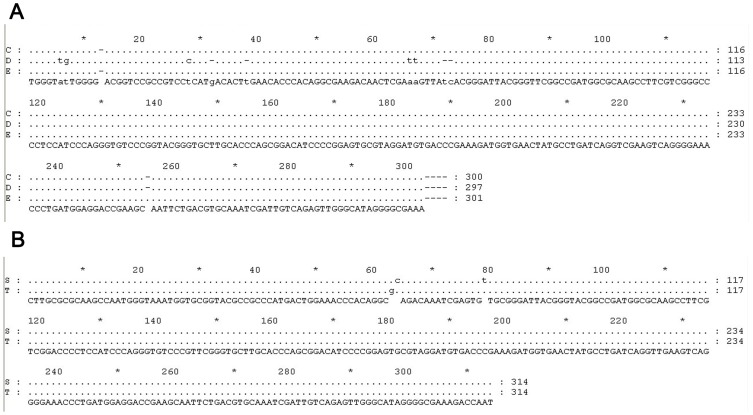
(a) Nucleotide alignment of *Anopheles culicifacies* species complex (GenBank database accession numbers KC494261, KC494262 & KC494264); (b) Nucleotide alignment of *Anopheles fluviatilis* species complex (GenBank database accession numbers KC 345546 & KC345547).

Table 5 showed that out of 3312 *An. culicifacies* assayed, 25 were positive for CSP of which, 14 were reactive for two polymorphs of *P. vivax* (7 VK 210 and 7 VK 247) and remaining for *P. falciparum*. The positive *An. culicifacies* were found during most part of the year except in January, February and March. They were caught from HD, Cattle shed (CS), PSC and LT (indoor/outdoor) and represented sibling species C, D and E (Figure 6a) (GenBank database accession numbers KC494261, KC494262 & KC494264). However, only one *An. culicifacies* (species C) was found positive from outdoor collection (LT). Additionally out of 67 *An. fluviatilis*, two were reactive for *P. falciparum* and represented sibling species T (Figure 6b) (GenBank database accession numbers KC345546, KC345547). Only one *An. fluviatilis* (sibling species S) was positive for CSP of *P. vivax* (VK 247).

**Table 5:** Vector incrimination of *Anopheles culicifacies* and *Anopheles fluviatilis* by ELISA from different sites in District Balaghat.

Type of collection	Site	Tested		+ve		Pv				Pf	
		An. cul.	An. fluv.	An. cul.	An. fluv.	An. cul.		An. fluv.		An. cul.	An. fluv.
						VK210	VK247	VK210	VK247		
Light Trap	Indoor	30	1	1	0	1	0	0	0	0	0
	Outdoor	59	0	1	0	1	0	0	0	0	0
MHD	HD	626	8	3	0	2	0	0	0	1	0
	CS	1440	45	12	0	2	3	0	0	7	0
PSC	HD	1157	13	8	3	1	4	0	1	3	2
Total		3312	67	25	3	7	7	0	1	11	2

+ve: Positive; Pv: *Plasmodium vivax*; Pf: *Plasmodium falciparum*; An. cul.: *Anopheles culicifacies*; An. fluv.: *Anopheles fluviatilis*; HD: Human dwelling; CS: Cattle shed; MHD: Man hour density; PSC: Pyrethrum spray catches.

Monitoring of insecticide susceptibility tests with *An. culicifacies* revealed that species is resistant to DDT and partially susceptible to malathion, lambda cyhalothrin and deltamethrin. The corrected percent mortality to DDT, malathion, deltamethrin and lambda cyhalothrin was 7, 82, 95 and 97% respectively. Cone bioassay test with alphacypermethrin showed 48% mortality on day 1 and 7.5% mortality on day 30.

## Discussion

The renewed effort to control malaria is founded on the recent preventive strategies and treatment options. In India the key interventions are IRS/ITNs/LLINs, RDTs and ACT. In the study area transmission occurs during most part of the year with a peak during post monsoon season. Overall, an increasing trend in malaria positivity was observed from 2010 to 2012 (chi-square for trend  =  663.55; P<0.0001). In this study >50% of the cases were reported in children less than 8 year and 31% cases were reported in older children between 8 to 14 years while only 18% cases above 14 years of age. The number of malaria cases and age group indicated that the risk for the age group under 8 years was 3.4 times greater than in adults suggesting a considerable degree of immunity in the adults.

More than 30% infants found infected with *P. falciparum* or *P. vivax* were less than 6 months of age. A review of age pattern of malaria revealed that as transmission increased, there was a shift of malaria towards younger age groups, regardless of seasonality [16], [17]. Overall, the most prevalent parasite was *P. falciparum* (80%) followed *by P. vivax*. It is interesting to point out that both *P. vivax* polymorphs, Pv 210 and Pv 247 were present almost equally while in North East India, VK 247 was predominant polymorphs [18]. Future studies are required to investigate if the same trend continues or both the polymorphs of *P. vivax* overlap [19]. *P. malariae* infections were recorded only in 2012 [4]. *P. malariae* is sustained at low infection rates among the sparse and mobile human population [20]. The high prevalence of *P. falciparum* and *P. vivax* malaria throughout the year with high sporozoite rate estimated for *P. falciparum* and *P. vivax* indicates that the vector control measures were inadequate and not effective in reducing adult vector density. Moreover, the environmental and climatological situations permit the continual breeding of vectors in permanent breeding sites. The sporozoite rate of *An. culicifacies* found in this study explains that *An. culicifacies* plays a major role in malaria transmission and *An. fluviatilis* played a role in enhancing *P. falciparum* transmission period along with *An. culicifacies* well beyond the wetter months. Moreover, for the first time *An. fluviatilis* sibling species T was found playing a major role in malaria transmission in forest villages along with species S in India.

The high infection rates with *P. falciparum*, *P. vivax*, *P. malariae* and mixed infection of *P. vivax*, *P. falciparum* and *P. malariae* suggest that vectors had a long life expectancy. Further, the patterns of infections are also determined by the occurrence and the ability of the vector species to be infected by different parasite species simultaneously [21]. IRS seemed to be ineffective in this area as there is a rapid decline of IRS insecticidal effectiveness within a month after spraying with alphacypermethrin. Moreover, IRS effectiveness depends on a high level of coverage. However, records revealed that in this area the amount of insecticide used for IRS varies from year to year. Actually the spray is carried out on the basis of epidemiological data collected from routine health facility surveillance record of the district malaria office. Data on number of malaria positives were based on village wise data pooled overall 12 months a year from all the PHCs of the district. Often this data is underestimated which resulted in limited spraying by the programme. Spraying also depends largely on the availability of insecticides in stock. Furthermore, *An. culicifacies* was also found positive in outdoor catches. The outdoor infectivity of *An. culicifacies* is of significance from a malaria control stand point, because the vector may avoid contact even with an effective insecticide sprayed inside the houses. Further research is required about the extra domiciliary transmission as the villagers frequently spent the night in the open, presumably providing a source of infection to the anophelines prevalent outdoors. During the survey, people also told that these bednets were never re-impregnated. Therefore, providing LLINs in such communities can remedy the short coming associated with ITNs. The World Health Organization also recommended the use of LLINs as these nets are designed to maintain their biological efficacy against vector mosquitoes for three years, obviating the need for regular insecticide treatment [22]. However, there are some weaknesses in the study. Because of the programmatic nature of intervention neither the spray campaigns are implemented rigorously and with high levels of house hold coverage nor a complete coverage of population with ITNs. Moreover, we could not check whether people are using bednet properly as about 85% nets were torn after 18 months. Entomological indices lacked human bait catches as the area is not safe. Thus entomological inoculation rate (EIR) could not be calculated.

## Conclusions

Tremendous progress has been made in some countries in reducing malaria related morbidity and many countries are striving for elimination of malaria [23], [24]. Amidst this process a major challenge is in forested areas which are also distributed and not approachable at least 2–3 months during raining season.

ITNs and IRS which are so effective in other parts of world [25] were not found effective in this area perhaps because of the outdoor life and forest based economy of the tribals [26]. Thus, there is a need to test the feasibility and effectiveness of other methods of control, for example - complete coverage of population with LLINs or with a new indoor residual insecticides which has longer residual life or both LLINs and IRS for effective vector control. Finally a well designed case control study may be taken up to evaluate the efficacy of these malaria interventions systematically.
